# Mobile Apps for Bipolar Disorder: A Systematic Review of Features and Content Quality

**DOI:** 10.2196/jmir.4581

**Published:** 2015-08-17

**Authors:** Jennifer Nicholas, Mark Erik Larsen, Judith Proudfoot, Helen Christensen

**Affiliations:** ^1^ Black Dog Institute University of New South Wales Sydney Australia; ^2^ School of Psychiatry Faculty of Medicine University of New South Wales Sydney Australia

**Keywords:** mobile applications, bipolar disorder, review, telemedicine

## Abstract

**Background:**

With continued increases in smartphone ownership, researchers and clinicians are investigating the use of this technology to enhance the management of chronic illnesses such as bipolar disorder (BD). Smartphones can be used to deliver interventions and psychoeducation, supplement treatment, and enhance therapeutic reach in BD, as apps are cost-effective, accessible, anonymous, and convenient. While the evidence-based development of BD apps is in its infancy, there has been an explosion of publicly available apps. However, the opportunity for mHealth to assist in the self-management of BD is only feasible if apps are of appropriate quality.

**Objective:**

Our aim was to identify the types of apps currently available for BD in the Google Play and iOS stores and to assess their features and the quality of their content.

**Methods:**

A systematic review framework was applied to the search, screening, and assessment of apps. We searched the Australian Google Play and iOS stores for English-language apps developed for people with BD. The comprehensiveness and quality of information was assessed against core psychoeducation principles and current BD treatment guidelines. Management tools were evaluated with reference to the best-practice resources for the specific area. General app features, and privacy and security were also assessed.

**Results:**

Of the 571 apps identified, 82 were included in the review. Of these, 32 apps provided information and the remaining 50 were management tools including screening and assessment (n=10), symptom monitoring (n=35), community support (n=4), and treatment (n=1). Not even a quarter of apps (18/82, 22%) addressed privacy and security by providing a privacy policy. Overall, apps providing information covered a third (4/11, 36%) of the core psychoeducation principles and even fewer (2/13, 15%) best-practice guidelines. Only a third (10/32, 31%) cited their information source. Neither comprehensiveness of psychoeducation information (*r*=-.11, *P*=.80) nor adherence to best-practice guidelines (*r*=-.02, *P*=.96) were significantly correlated with average user ratings. Symptom monitoring apps generally failed to monitor critical information such as medication (20/35, 57%) and sleep (18/35, 51%), and the majority of self-assessment apps did not use validated screening measures (6/10, 60%).

**Conclusions:**

In general, the content of currently available apps for BD is not in line with practice guidelines or established self-management principles. Apps also fail to provide important information to help users assess their quality, with most lacking source citation and a privacy policy. Therefore, both consumers and clinicians should exercise caution with app selection. While mHealth offers great opportunities for the development of quality evidence-based mobile interventions, new frameworks for mobile mental health research are needed to ensure the timely availability of evidence-based apps to the public.

## Introduction

Symptom monitoring is a fundamental component of the self-management of bipolar disorder (BD) that benefits from being performed in real-time and under natural conditions [1]. With mobile phone penetration reaching 97% worldwide, and smartphones accounting for the majority of phone sales in 2014 [2], researchers and clinicians are investigating the use of smartphone technology to enhance the management of chronic illnesses such as BD. As smartphones are personal devices that are carried on the user, mobile apps are a perfect platform for self-management. Smartphones can also be used to deliver interventions and psychoeducation, supplement treatment, and enhance therapeutic reach in BD, as apps are cost-effective, accessible, anonymous, and convenient.

A study by Proudfoot et al [3] found that consumers hold positive attitudes towards evidence-based mental health apps, with 76% of survey responders indicating that they would be interested in using their smartphones for monitoring and self-management of mental health. This acceptability has also been shown in mobile programs for BD, with personal digital assistant [4,5] and short message service based interventions [1] proving effective and acceptable to consumers. Similar programs are now being developed for smartphones. For instance, Wenze et al [6] are developing an expanded smartphone version of their successful PDA-based “Increasing Adherence in Bipolar Disorder” intervention, which will incorporate psychoeducation and Cognitive Behavioral Therapy (CBT) sessions using smartphones alongside in-person treatment to address medication adherence. A smartphone app based on Interpersonal and Social Rhythms Therapy (IPSRT) augmented with objective data from phone sensors is also in production. This app, called “MoodRhythm”, aims to stabilize daily routines and rhythms to prevent episode-onset [7]. Also investigating the potential use of objective data alongside self-report, the monitoring, treatment, and prediction of bipolar disorder episodes (MONARCA) study app attempts to validate objective indicators of affect change by investigating correlations with user-inputted and clinician mood ratings [8]. Additionally, Grünerbl et al [9] are investigating extending this research to enable the app to identify mood state changes based solely on objective sensor data. However, to our knowledge, there are currently no apps available for people with BD that have been subject to research evaluation to determine effectiveness.

Previous reviews have found that apps currently available to consumers for other health conditions are unlikely to be supported by research data, nor be developed with reference to evidence-based practice. For example, an investigation of the content of smoking cessation apps found that apps had low concordance with guidelines, with an average guideline adherence score of 7.8 out of a possible total of 60 [10]. Examining other chronic conditions with a strong self-management focus, a review of apps for asthma self-management determined that only 5% of those providing information were comprehensive, while 44% made recommendations that were not in line with treatment guidelines [11]. Similarly, a review of apps for human immunodeficiency virus / sexually transmitted disease prevention and care found that only 11% of assessed apps covered all literature-driven prevention areas [12]. Moreover, Donker et al [13] performed a systematic review of apps for mental health and noted the striking disparity between the number of apps available and the number of scientific articles assessing the evidence base of mental health apps. Similarly, a simultaneous review of health-related apps and research literature for a number of conditions noted the contrast between the number of research publications regarding the use of mobile technology to manage the investigated illnesses and the profusion of available apps [14].

An examination of the Google Play and Apple iOS app stores indicates that there is an abundance of publically available apps for BD despite the limited published research. Therefore, there is little information accessible to consumers about the quality of available apps beyond the user “star” rating system and user reviews. However, the popularity of an app is not necessarily a reliable indicator of its quality, effectiveness, or evidence base. The opportunity for mHealth to assist in the self-management of BD is feasible only if apps are of appropriate quality, and therefore, the aim of the current study was to identify the types of self-management apps available in the Google Play and iOS app stores and to assess the comprehensiveness and quality of their content for BD.

## Methods

### Search Criteria and Selection

A systematic review framework was applied to the search, screening, and assessment of apps.

Search terms to identify apps developed specifically for BD were derived through a preliminary search of the Google Play store. Relevant synonyms and layperson alternatives to the identified terms were determined and also included in the search. Layperson alternatives were included because our aim was to assess apps that were specifically designed for consumers with BD, a group that may not use the technical terms of the disorder. In this way, the following search terms were identified: bipolar, bi-polar, “manic depression”, “mood swings”, “mania” AND “mood”, cyclothymia, and cyclothymic. On July 16, 2014, these terms were used to identify publically accessible apps for BD in the Australian Google Play and iOS app stores. Android apps were identified by a search of the Google Play store via the Web interface, while iOS apps were identified via the iTunes/iOS search application programming interface.

Details of apps identified from the app stores were extracted from the search results including app name, description, and price. Duplicate apps that were retrieved by multiple search terms on the same device platform were removed. Conversely, apps with different versions (eg, free/paid) or which appeared across different platforms were retained for comparison. Two reviewers (JN and ML) independently screened the app store description of each app. Apps were included in the review if the following criteria were met: (1) developed for BD, (2) aimed at consumers and/or caregivers, (3) available for download through official Android/iOS app stores, and (4) available in English. Discrepancies were identified and discussed until consensus was achieved.

### App Assessment

Apps meeting the inclusion criteria were downloaded onto either a Samsung Galaxy S4 mini (Android version 4.2.2) or an iPhone 5s (iOS version 7.1) for complete assessment. Two reviewers (JN and ML) performed the assessment of apps using a standard data extraction form.

Descriptive characteristics related to the following features were extracted: (1) app accessibility, including platform, price, need for network connectivity, number of downloads, and average user-scored star rating for the app’s entire history, (2) primary function, such as app store classification, app function (see below), and target audience, (3) app source, including provider type and details, crisis information, disclaimer presence, and app update schedule, and (4) privacy and security, presence of an accessible privacy policy, and the ability to password-protect data.

The function of each app was determined through use of the app and classified as providing either information or self-management tools. Additional assessment was performed based on the identified function. Apps containing information about BD were assessed according to the comprehensiveness of its psychoeducation information and the quality of the information, as reflected by its concordance with evidence-based guidelines. The comprehensiveness of BD psychoeducational information was assessed according to 11 fundamental topics of face-to-face psychoeducation interventions [15]. Core statements were derived from the main topics and goals of psychoeducation with reference to Colom and Vieta’s Psychoeducational Manual for Bipolar Disorder [15] (Table 1). A clinical psychologist with experience with BD then reviewed the statements for accuracy and completeness.

**Table 1:** Core components of psychoeducation for BD.^a^

Topic	Criteria
1. Facts about the nature of BD.	States that BD is biological in nature but interacts with environmental factors (diathesis-stress model).
	States that BD is chronic and recurrent in nature and has a cyclic course.
2. Information on common symptoms of each phase of the disorder.	States common symptoms of both (hypo)mania and depression.
	States that risk of suicide is associated with BD.
3. Treatment options for each illness phase.	Outlines available pharmacotherapy for each illness phase: depression, mania, and prophylaxis.
	Mentions psychotherapy as a treatment option for BD.
4. Treatment adherence, withdrawal, and side effects.	States the importance of treatment adherence and states that risk of episode relapse is associated with abandonment of treatment.
5. Substance use in BD.	States that psychoactive substances may trigger episodes.
6. Identification of episode warning signs (EWS).	States common EWS of (hypo)mania and depression.
	States that EWS vary between people and indicates the importance of identifying personal episode warning signs.
7. Support networks and the role of support people or caregivers.	Describes a support person as someone who is close to the patient, aware of their BD, and knowledgeable about the disorder.
	States that a support network can assist in early detection of episodes.
8. The role of an action plan.	States the importance of having an action plan that provides a guide to stay well when episode EWS are detected.
	States common strategies that help prevent episodes once EWS are detected.
9. The importance of routine.	States that regular habits, including sleep, are of importance in BD.
	States that regular schedules and better structuring of activities are key in BD management.
10. Information on stress management and problem solving.	States that stress plays an important role in episode relapse.
	States there are tools that help manage stress and anxiety.
11. Episode risk-factors/triggers.	States common external factors that contribute to episode relapse.

^a^Topics based on Colom and Vieta’s psychoeducation for BD manual [15].

The quality of BD information was assessed as the degree of concordance with the most recent treatment guidelines [16-19] and prominent meta-analyses and reviews of evidence-based treatment for the acute phases of the disorder [20,21]. Thirteen statements were derived from treatment guidelines and meta-analyses for use as quality indicators in the app review (Table 2; [16-24]). A psychiatrist previously involved in the development of the Australian and New Zealand Clinical Practice Guidelines for the Treatment of Bipolar Disorder [22] then assessed and adjusted the statements for accuracy and comprehensiveness. Comprehensiveness and quality of information were calculated as a score out of 11 and 13 respectively, indicating how many of the core psychoeducation and guideline-derived statements were covered by the app’s information.

Apps providing common tools for the management of BD were assessed against best-practice resources for their purpose. Screening and assessment apps were assessed according to whether the app used a validated BD screening questionnaire or how closely it reflected current diagnostic criteria [25,26]. Symptom monitoring apps were evaluated according to their similarity to well-known paper-and-pencil monitoring tools [27,28]. Treatment apps for BD were assessed against statements derived from the core goals of three commonly used therapies for the disorder: CBT [29], IPSRT [30], and Family-Focused Therapy (FFT) [31]. As with statements used to assess the comprehensiveness and quality of information, treatment statements were reviewed by a clinical psychologist with experience with BD. Community support apps, which provided access to BD-orientated discussion boards, were assessed based on their methods of communication, and the level of moderation or oversight.

**Table 2:** App quality assessment statements derived from BD treatment guidelines and meta-analyses.

Statement	Associated guideline
1. Initiation of an atypical antipsychotic and/or mood stabilizer for the treatment of acute mania.	“Efficacy of lithium and divalproex is well established…substantial RCT data support atypical antipsychotic monotherapy with olanzapine, risperidone ER, quetiapine, ziprasidone, and aripiprazole for the first-line treatment of acute mania” Yatham et al, 2013, pp 4, 6 [19]
	“Overall, risperidone, olanzapine, and haloperidol seem to be the most effective evidence-based options for the treatment of manic episodes” Cipriani et al, 2011, pp 1314 [20]
2. Use of an atypical antipsychotic or mood stabilizer, with or without an antidepressant, for the treatment of bipolar depression.	“Lithium, lamotrigine, quetiapine…monotherapies, as well as lithium or divalproex plus selective serotonin reuptake inhibitor, olanzapine plus SSRI…recommended as first-line choices for bipolar depression” Yatham et al, 2013, pp 9 [19]
	“Adjunctive antidepressants may be used for an acute bipolar I or II depressive episode when there is a history of previous positive response to antidepressants” Pacchiarotti et al, 2013, pp 1253 [21]
3. Antidepressant subtypes tricyclic antidepressants and serotonin-norepinephrine reuptake inhibitors (SNRIs) are more likely to cause switching than serotonin-specific reuptake inhibitors (SSRIs).	“The risk of mood switching is considered to be…somewhat greater with tri-and tetracyclics (and perhaps some SNRIs) than with most modern antidepressants” Pacchiarotti et al, 2013, pp 1256 [21]
	“monotherapy with some antidepressants, especially tricyclics, without an accompanying mood stabilizer, however, may be associated with an increased rate of treatment emergent affective switches (TEAS)” Grunze et al, 2010, pp 92 [17]
4. Lithium, an atypical antipsychotic, or lamotrigine (where depression predominates) for maintenance treatment of BD.	“Lithium, divalproex, olanzapine, and quetiapine, as well as lamotrigine (primarily for preventing depression)…continue to be first-line monotherapy options for maintenance treatment of BD” Yatham et al, 2013, pp 14 [19]
	“Lithium, olanzapine or valproate should be considered for long-term treatment of bipolar disorder” NCCMH, 2006, pp 5 [23]
5. Change monotherapy or use combination therapy for treatment resistance.	“No response after 2 weeks, switch to another first choice medication, in severe mania, consider combination” Grunze et al, 2009, pp 104 [16]
	“If the patient has frequent relapses, or symptoms continue to cause functional impairment, switching to an alternative monotherapy or adding a second prophylactic agent should be considered.” NCCMH, 2006, pp 5 [23]
6. The use of electroconvulsive therapy (ECT) for treatment resistant acute symptoms (particularly depression, but also mania).	“Especially in very severe and psychotic depression, or in depression with severe psychomotor retardation, ECT has a major role” Grunze et al, 2010, pp 100 [17]
	“ECT is recommended for bipolar depression after an antidepressant trial has failed” RANZCP, 2004, pp 288 [22]
	“ECT is still a valuable last resource in severe delirious mania which is otherwise treatment refractory” Grunze et al, 2009, pp 102 [16]
7. Careful monitoring of blood levels is required where those correlate with treatment response (eg, lithium, valproate).	“Plasma concentrations need to be checked on a frequent and regular basis until equilibrium in the therapeutic range as been achieved and thereafter. It is recommended to check every 3-6 months” Grunze et al, 2013, pp 186 [18]
	“lithium is up titrated in small steps guided by individual experience and plasma level monitoring” Grunze et al, 2013, pp 186 [18]
8. Careful monitoring of potential physical complications or side effects of treatments is required (eg, kidney, thyroid, and calcium with lithium; glucose and lipids with antipsychotics).	“Renal and thyroid function should also be checked regularly, every 6-12 months depending on risks” Grunze et al, 2013, pp 186 [18]
	“Complete medication and laboratory investigations should be performed at baseline, with ongoing monitoring for weight changes and adverse effects of medication” Yatham et al, 2013, pp 29 [19]
9. Women informed about ensuring that their medications are safe to take during breastfeeding and pregnancy.	“Important that women with bipolar disorder receive education early in the course of illness about the effects of mood stabilizing and other medications on contraceptive effectiveness, as well as the need to plan medication management during pregnancy and the postpartum period” Yatham et al, 2005, pp 33 [24]
10. Seek medical professional advice and/or a second opinion in diagnosis of BD in children, due to the controversy in this area.	“The presentation and diagnosis of BD in children and adolescents remains controversial…diagnostic criteria for BD may not be systematically applied in some clinical settings.” Yatham et al, 2013, pp 19 [19]
11. Outlines difficulties in the treatment of rapid cycling BD.	“Rapid cycling…is associated with greater severity of illness on a number of clinical measures” Yatham et al, 2005, pp 30 [24]
	“The prophylactic use of lithium in rapid cycling patients has been discouraged for a long time based on the observation of insufficient acute and prophylactic efficacy in these patients” Grunze et al, 2013, pp 184 [18]
12. Optimal treatment for most patients with BD will include psychological treatment as well as medication.	“When used as adjuncts to pharmacotherapy, psychosocial interventions…have demonstrated significant benefits, both in the treatment of acute depressive episodes and also as long-term maintenance treatment…providing psychological treatments—and, in particular, brief psychoeducation, which has been demonstrated to be as effective as CBT at much lower cost—is an essential aspect of managing patients with BD” Yatham et al, 2013, pp 4 [19]
	“The primary long-term treatments are pharmacological, but psychological and psychosocial interventions have an important part to play” NCCMH, 2006, pp 33 [23]
13. Indicates that most patients benefit considerably from treatment for their BD.	“The advent of these therapies, both drug and psychological, means that the majority of patients with this recurrent and disabling condition may be effectively treated” RANZCP, 2004, pp 299 [22]

### Data Analysis

Descriptive statistics were used to summarize the results of the app assessment. Pearson product-moment correlation was performed to examine relationships between comprehensiveness and quality of information, and the average user rating of an app. Mann-Whitney U tests were performed to examine differences in comprehensiveness and quality of information by app price. Statistical significance was set at *P*<.05.

## Results

**Figure 1:**
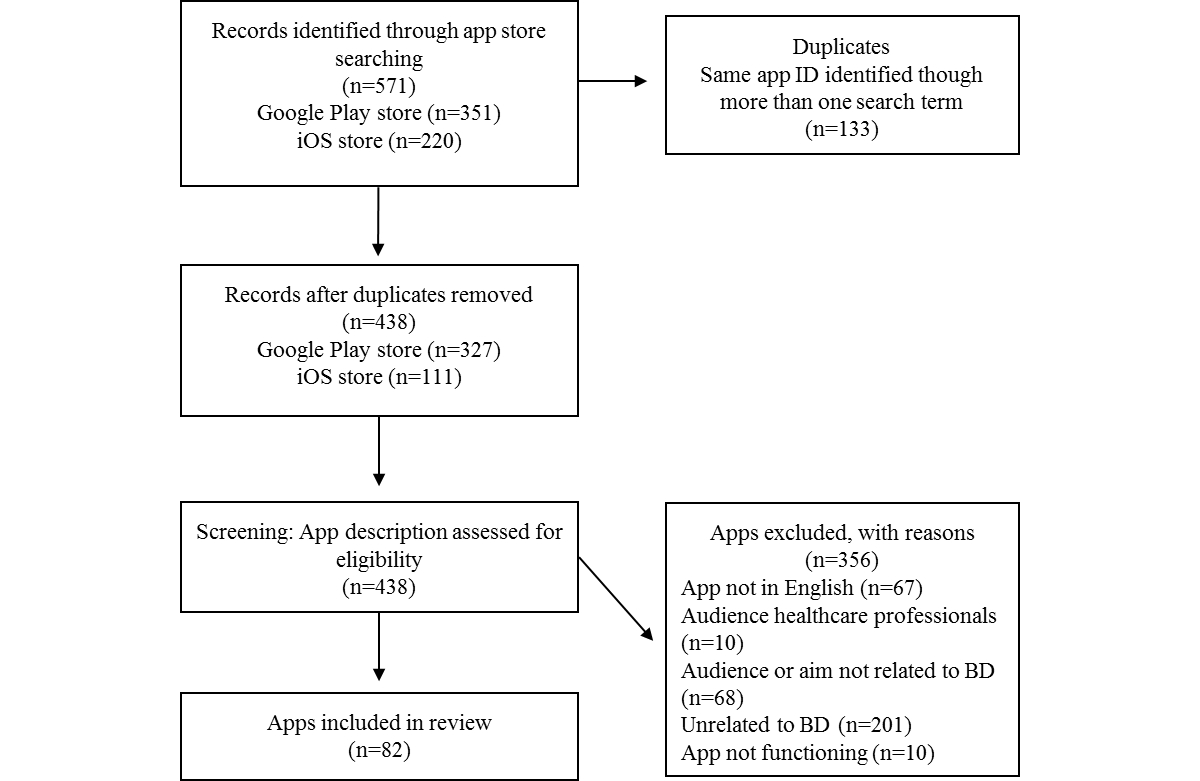
Flow diagram illustrating the exclusion of apps at various stages of the study.

Google Play and iOS app store searches identified 571 potential apps, of which 133 were removed as duplicates (identified on the same platform through more than one search term). Of the remaining 438 apps, 82 met inclusion criteria (see Multimedia Appendix 1 for a list of included apps). Details about the inclusion and exclusion of apps are provided in Figure 1.

### Description

#### Accessibility

Of the 82 included apps, 59 were available on the Android platform, 23 were available on iOS devices, and ten had versions for both platforms. Examining costs, 49 apps were free, and the median cost of paid apps was AU$1.70, with a minimum price of AU$.99 and a maximum of AU$16.99 (mean AU$3.05). Over half (n=48) did not require an active Internet connection after installation, 20 would not function in the absence of an Internet connection, and 14 needed connectivity for some features. Only the Google Play store reported the number of downloads of each app to date, indicated by a range. The median number of downloads was between 1000 and 5000 (information available for 53 of the apps). Fourteen of these 53 apps had been installed less than 100 times. The app store descriptions of 28 apps indicated that an insufficient number of users had rated the app over its lifetime to provide an average user rating. The mean average user rating of the remaining 54 apps was 3.5 out of a possible 5.

#### Primary Function

On the app stores, apps for BD were classified as health and fitness (n=48), medical (n=15), lifestyle (n=6), books (n=3), social (n=2), and entertainment (n=1), with genre information unavailable for seven apps. Thirty-one apps provided written information about the disorder, and one used animation to raise awareness. The remaining 50 apps provided tools for the management of BD and included ten screening and assessment tests, 35 symptom monitoring apps, four community support discussion boards, and one treatment app. No apps performed multiple functions.

For 78 apps, the function of the app was clearly stated in the app store description. Apps with unclear descriptions either introduced the disorder without explaining the role of the app, were unclear between two distinct functions, or provided information for an unrelated app. Forty-nine apps were developed for use by patients, with four apps specifically designed for caregivers, 14 for the general public, and 15 for both patients and caregivers. According to the app store descriptions, no apps were developed for a specific BD clinical subgroup.

#### App Source

As far as we can discern, none of the 82 apps assessed had been subject to research evaluation. No apps cited published material about the feasibility or effectiveness of the app, either within the app or the app store description. Furthermore, there was a lack of Google Scholar results upon a search of app names.

The majority of apps were developed by commercial providers (n=48) or private individuals (n=20), followed by institutions (n=3), other types of providers (n=6) including, clinical groups and not-for-profit organizations, and unspecified (n=5). The app provider’s name was included in 46 apps, of which 30 also included contact details; eight apps included only provider contact details. A disclaimer outlining that the app was not a replacement for consultation with a medical professional was provided in 23 of the 82 apps, including seven screening and assessment apps, and eight information apps. A statement providing information and contact details of crisis resources was provided in only nine apps for BD. A quarter of apps (n=19) had been updated within the last 6 months, with half of the apps updated within the last 17 months.

#### Privacy and Confidentiality

Privacy policies were available, either in the app or as a link from the app store description, for 18 of the 82 apps. Of the 50 apps able to record personal data (screening and assessment, symptom monitoring, community support, and treatment apps), 12 had a privacy policy. An account or password was needed to access these data for 19 of these apps.

### Information

#### Overview

Apps containing information were more readily available on the Android (n=29) compared to the iOS (n=3) platform. Of the 32 apps that provided health information, seven presented general health news stories about a variety of conditions, including BD, and a further six provided no disorder-specific information to assess. The remaining 19 information apps were assessed for comprehensiveness and quality.

Of these 19 apps, all contained in-app information content, and four linked directly to additional external information. In all cases, external material was referenced; however, just four credited the source of their in-app information. Although only three of the apps were classified on the app stores as books, the assessment revealed 12 information apps were repurposed ebooks. Although unattributed, many shared the same ebook content, with nine apps having the same information content as another app, but with differences in user interface layout, connectivity reliance, or wording.

#### Comprehensiveness of Bipolar Disorder Psychoeducational Information

The comprehensiveness of the psychoeducation information presented in the apps is reported below (Table 3). Overall, apps covered an average of four of the 11 psychoeducation statements (SD 3.0). Two statements, concerning the importance of treatment adherence and the development of action plans as a guide to stay well, were not addressed by any app. Five of the apps contained no BD specific psychoeducation information in line with best practice, while the most comprehensive apps covered seven statements (seven apps). These seven apps were reproductions of the same content, although uncited.

**Table 3:** Comprehensiveness of psychoeducation topics covered by BD information apps.

Topic	Apps covering topic, n (%)
1. Facts about the nature of BD.	11 (58)
2. Information on common symptoms of each phase of the disorder.	14 (74)
3. Treatment options for each illness phase.	5 (26)
4. Treatment adherence, withdrawal, and side effects.	0 (0)
5. Substance use in BD.	8 (42)
6. Identification of episode early warning signs.	7 (37)
7. Support networks and the role of support people or caregivers.	8 (42)
8. The role of an action plan.	0 (0)
9. The importance of routine.	8 (42)
10. Information on stress management and problem solving.	8 (42)
11. Episode risk-factors/triggers.	3 (16)

#### Quality of BD information: Concordance With Evidence-Based Practice

Of the 13 evidence-based statements extracted from treatment guidelines for BD, an average of two statements were covered by apps (SD 2.3; Table 4). Only three apps provided information aligned with more than three guidelines, covering four, seven, and eight statements, and six apps addressed no statements. The app that covered seven statements comprised 50 chapters, with no logical structure and repeated unreferenced information. In contrast, the app that addressed eight statements contained information structured into six concise sections and referenced NHS Choices [32].

**Table 4:** Quality of BD app information: concordance to BD treatment guidelines.

Statement	Apps covering topic, n (%)
1. Initiation of an atypical antipsychotic and/or mood stabilizer for the treatment of acute mania.	3 (16)
2. The use of an atypical antipsychotic or mood stabilizer, with or without an antidepressant, for the treatment of bipolar depression.	2 (11)
3. Antidepressant subtypes tricyclic antidepressants and SNRIs are more likely to cause switching than SSRIs.	0 (0)
4. Lithium, an atypical antipsychotic, or lamotrigine (where depression predominates) for maintenance treatment of BD.	4 (21)
5. Change monotherapy or use combination therapy for treatment resistance.	1 (5)
6. The use of ECT for treatment resistant acute symptoms (particularly depression, but also mania).	9 (47)
7. Careful monitoring of blood levels is required where those correlate with treatment response (eg, lithium, valproate).	2 (11)
8. Careful monitoring of potential physical complications or side effects of treatments is required (eg, kidney, thyroid, and calcium with lithium; glucose and lipids with antipsychotics).	1 (5)
9. Women informed about ensuring that their medications are safe to take during breastfeeding and pregnancy.	1 (5)
10. Seek medical professional advice and/or a second opinion in diagnosis of BD in children, due to the controversy in this area.	1 (5)
11. Outlines difficulties in the treatment of rapid cycling BD.	1 (5)
12. Optimal treatment for most patients with BD will include psychological treatment as well as medication.	10 (53)
13. Most patients benefit considerably from treatment for their BD.	10 (53)

Across the apps that provided information about BD, four contained incorrect information, three of which incorrectly differentiated the different types of BD. Two contained critically wrong information; the first suggested, “take a shot of hard liquor a [sic] hour before bed” (app ID 404) to assist with sleep during a manic episode. The second incorrectly informed users about the types of BD and indicated that BD was contagious “sometimes [BD] can transfer to another relative if they spend too much time with you and listen to your depressive life” (app ID 28).

#### User Ratings

Neither comprehensiveness of psychoeducation information (*r*=-.11, *P*=.80) nor information quality (*r*=-.02, *P*=.96) were significantly correlated with average user ratings. A Mann-Whitney U test also revealed no significant difference in comprehensiveness (*U*=30.5, *Z*=-1.2, *P*=.22) or quality (*U*=42.5, *Z*=-.21, *P*=.83) between free and paid apps.

### Tools

#### Screening and Assessment

Ten apps offered self-assessments to screen for BD, of which three were for children. Only three cited the source of the test, two of which used a validated screening measure (the M3 checklist) [33]. Two additional apps used the Mood Disorder Questionnaire [34] without attribution. In total, only four of the 10 screening apps used validated measures, and six of the tests screened for only manic symptoms.

Of the apps that asked about symptoms of depression, three had a duty-of-care message at the end of the test, referring the user to clinical support if questions about suicidal ideation were answered positively. Upon informing the user they had screened positively for BD, eight apps recommended visiting a health care professional, and two had the option for users to share the result on social media.

#### Symptom Monitoring

In total, 35 apps were symptom monitoring tools, which aim to assist users with tracking the symptoms of their BD. The number of apps monitoring mood, medication, sleep, and other symptoms is shown in Table 5. A mood scale designed for BD was defined as a continuous scale with “depressed” and “manic” at the extremes. Other scales used to monitor mood included using different emoticons to represent emotions (n=10), rating mood on a generic scale (n=8), or had users select from or rate a list of moods (n=4). Thirty apps monitored factors not commonly included on established mood charts, with energy levels and anxiety the most common additions. These tracking options were customizable in nine apps, allowing users to monitor factors specifically relevant to their mood states. General customization of other features such as esthetics, profiles, and data reports was possible in 21 apps.

**Table 5:** Symptoms monitored by symptom monitoring apps for BD.

Factors monitored	n (%)
Mood	34 (97)
Mood on scale designed for BD	12 (34)
Medication	15 (43)
Sleep	17 (49)
Functioning	3 (9)
Section for free-notes	32 (91)
Mixed episodes or mood switches	2 (6)
Menstruation	5 (14)
Other	30 (86)

Alerts reminding users to track their mood were available in 22 of the 35 monitoring apps. Of these, 13 allowed users to designate a reminder time, six asked users to designate how many times per day to be reminded, and three had options for both. Over a sampling period of 3 days, seven apps failed to notify the user as directed.

None of the 35 monitoring apps had a duty-of-care alert, that is, a message suggesting users contact a health care provider. This was tested by logging 3 consecutive days of severely depressed mood and indicating suicidal ideation in free-text notes. Inputted monitoring data was presented graphically by 30 apps, could be exported from 20, and shared via social media by seven. Back-up of personal monitoring data was available in only six apps. No app used the sensor capabilities of the smartphone to enhance monitoring data beyond what is possible with paper-and-pencil resources, or as an attempt to validate subjective mood reporting with objective data.

#### Treatment

There was only one treatment app, which delivered a CBT intervention, although it had not been specifically developed for BD. An analysis of treatment content against common treatment features of CBT, IPSRT, and FFT confirmed that the app was based on CBT principles, with three of the six CBT items endorsed, and none related to other therapies. However, the source or evidence base of the CBT presented in the app was not referenced.

#### Community Support

Four community apps gave users access to BD-orientated discussion boards, where members sought information and support. App communities were for consumers and family/caregivers and allowed communication via forum posts and private messages. Site owners for each app monitored forum post communication.

## Discussion

### Principle Findings and Comparison With Prior Work

This assessment of apps for BD indicates that most currently available apps do not reference clinical practice guidelines, standard psychoeducation information, or established self-management tools. Apps were available for a variety of uses including disorder information (38%), and management tools such as screening and assessment (12%), symptom monitoring (43%), and community support (5%). Interestingly, no apps combined these functions to provide both information and self-management tools. Furthermore, while all the assessed apps mentioned BD in their app store description, the content evaluation indicated few had been designed specifically for the disorder. For example, only a third of mood monitoring apps provided a BD specific mood scale, with the remaining apps using scales inadequate for the population. Furthermore, merely half of the apps claiming to provide information about BD actually provided disorder-specific information to be assessed for comprehensiveness and quality.

Disappointingly, apps that did provide BD specific information had low levels of adherence to quality assessment criteria, neither comprehensively addressing main psychoeducation domains, nor endorsing evidence-based practice guidelines. The app that correctly addressed the most treatment guideline criteria was Your MD’s Symptom Checker (app ID 39), which referenced NHS Choices [32], and presented information concisely and coherently (only BD information was assessed). Overall, comprehensiveness and quality scores were low and not correlated with average user ratings, confirming that an app’s popularity is not an accurate gauge of its content. However, with only a third of information apps citing their information source, users are denied other important information by which to make informed judgments about an app’s robustness and credibility.

Apps that offered tools to assist with the management of BD were also inconsistent in quality. Few screening and assessment apps citied the source of the test; additionally, a Google search of each app’s assessment questions revealed that less than half used validated screening measures. Only one treatment app was included in the review, and while it covered half of the CBT treatment domains, it was not specifically designed for the needs of people with BD. Symptom monitoring was the most prevalent self-management tool offered by apps. However, only a third allowed users to track all three symptoms standard in traditional monitoring tools: mood, sleep, and medication. The high proportion of monitoring apps that did not feature a notification or alert function to remind users to input daily mood data was unexpected. As the ability to remind users to complete monitoring in situ is a major advantage of this technology, it is surprising that more than a third of monitoring apps did not utilize this function, and a further seven did not ensure the feature functioned as intended. It was also an unexpected finding that no symptom monitoring apps used the smartphone’s sensors to supplement the user-inputted monitoring data. Torous et al [35] found that all published articles regarding apps for bipolar disorder focused on the use of passive sensor-enabled data collection with supplemental or no user input. This is another example of the disconnect between emerging research in this area, and apps currently available for the disorder.

These results are consistent with findings of reviews assessing apps of other heath domains. Unfortunately, many of these reviews examine quality using the information provided in the app store description rather than assessing app content against evidence-based clinical guidelines [36]. However, those that do assess content have found similarly low endorsement of quality criteria. Huckvale et al [11] assessed asthma information against evidence-based asthma treatment guidelines and reported that the most endorsed guideline was cited by only 32% of apps. A similarly low concordance with guidelines was found in a review of smoking cessation apps [10]. While a recent review of depression apps did not assess content, it highlighted another difficulty faced by users of health apps. Shen et al [37] reported that a search for depression on the five major app stores yielded three times more non-related apps than depression apps [37]. In the current study, 78% of apps were excluded due to not being related to BD or not having a BD specific aim. This suggests an app’s quality is not the only challenge consumers face in identifying appropriate apps, as irrelevant alternatives dominate search results. Together, these findings suggest that the results of the current study reflect a wider problem of app availability and quality across health conditions.

Rarely considered, the lack of privacy policies made available to app users was another area in which apps developed for BD fell short of optimal standards. A recent review by Dehling et al of information security and privacy of mobile health apps characterized apps that had access to mental health information as high sensitivity [38]. The review identified health monitors, state of health tests, treatment reminders, and health records, which included disease management tools, as the highest privacy and security risks [38]. However, only a quarter of the assessed BD self-management tools, which would fall into the above categories, provided a statement indicating how data would be protected, stored, and shared. Sunyaev et al [39] reported a similar absence of mHealth privacy policy availability. Their assessment of health apps found that only 30.5% provided privacy policies, two-thirds of which did not address the app specifically [39]. This represents a problematic lack of transparent reporting of how apps handle personal and mental health-related data. As the use of apps in health care becomes more common, the topic of information privacy will continue to grow in relevance, as privacy and security consistently feature as major considerations of users [3,38]. Therefore, the time to ensure structures are in place to address privacy and security is now.

While apps asked users to input personal health data, very few responded to indications that users were unwell. In fact, only three apps responded to users indicating severe extremes of mood or suicidal ideation. A lack of response to severity of recorded symptoms was also noted by Huckvale et al [11] in asthma management tools. Although both psychoeducation and clinical guidelines emphasize the importance of the ability to identify and respond to indications of mood change, few apps assisted users by informing them of such changes. This identification and response to indicators of change in mood forms the basis of action plans or stay well plans. Action plans are a common feature of psychoeducation [15] and combine information about early warning signs and factors that may ameliorate them into a plan that aims to prevent episode relapse. Action plans are also a common self-management tool used by people with BD to stay well [40,41]. A cohort of individuals that had not experienced an episode of BD for more than 2 years were interviewed about their strategies for staying well [41]. All participants discussed the importance of a tailored and revised stay well plan, which identified triggers and early warning signs and designated strategies to intervene. However, no information apps mentioned action plans, and no self-management tools assisted users in the development of an action plan. Together with the lack of response to user input, this represents a missed opportunity for the facilitation of important self-management practices by mobile technology.

### Limitations

The current study is not without its limitations. A possible limitation is that the apps assessed were those available through the Australian Google Play and iOS app stores, and app availability may differ globally. This may affect the generalizability of results to apps available in other locations. However, a search of the Australian, British, and American iTunes app stores using the primary search term “bipolar” indicated that results were remarkably comparable. In total, 97% of apps were available in all three regions, with only three apps exclusively available in the American store. Therefore, at least in English-speaking countries, apps appear to be universally available, and this review provides a representative overview of the apps available internationally. A second possible limitation is that while this study is novel in that it provides, to our knowledge, the first assessment of apps for BD in respect to clinical guidelines, psychoeducation principles, and self-management tools, it does so for a snapshot of apps available when the search was performed in July 2014. As such, with changing nature of the Google Play and iOS app stores, the marketplace of apps for BD will differ by time of publication.

### Conclusions

With the exponential increase in the use of smartphones, there has been an increased interest in the use of apps for information dissemination and disease management. However, this study’s findings highlight important shortcomings in current app marketplace offerings for BD, suggesting that apps are developed independently of research data, and without reference to best practice clinical guidelines. These results indicate that clinicians looking to recommend apps to supplement treatment should exercise caution with app selection and that policy makers and the research community need to consider ways of assuring app quality. There is an opportunity for mental health research to develop quality evidence-based mobile interventions that assist with the management of BD. However, with the domains of research and technology currently moving at different paces, new frameworks for mobile mental health research are needed to prevent a lag in availability and to ensure that evidence-based apps are available to consumers.

## Appendix

Multimedia Appendix 1 List of apps included in the review.

## Abbreviations

BD: bipolar disorder
CBT: cognitive behavioral therapy
ECT: electroconvulsive therapy
EWS: episode warning signs
IPSRT: interpersonal and social rhythms therapy
FFT: family-focused therapy
SNRI: serotonin-norepinephrine reuptake inhibitors
SSRI: serotonin-specific reuptake inhibitors
