# Supplementary material for: Mobile Apps for Bipolar Disorder: A Systematic Review of Features and Content Quality
Source: J Med Internet Res. 2015 Aug 17;17(8):e198. doi: 10.2196/jmir.4581 (PMC4642376; doi:10.2196/jmir.4581)
Supplement: Multimedia Appendix 1 [file jmir_v17i8e198_app1.pdf]

Multimedia Appendix 1: List of apps included in the review.

App ID	Title	Developer	Function	Price	Version
1	Recovery International	Brian Corson	Information	$0.00	
6	eMoods Bipolar Mood Tracker	Yottaram LLC	Symptom monitoring	$0.00	
7	Bipolar Treatment	Karl Evans	Information	$0.00	130110<br>14
8	Bipolar Test	Consurgo	Screening and assessment	$0.00	1.5
9	Bipolar Tracker	William Alexander	Screening and assessment	$0.00	
10	Bipolar Connect	Alliance Health Network	Community support	$0.00	
11	Bipolar	Kindle Trove Apps	Information	$0.00	4.0
12	Mood Chart Bipolar Depression	Monitor My Health, LLC	Symptom monitoring	$0.00	1.01
14	Bipolar Disorder Treatment	InfoApps24	Information	$0.00	
15	Bipolar Disorder Uncovered	KoolAppz	Information	$1.29	1.0
18	Bipolar Disorder	Deep Powder Software	Information	$3.00	1.0
19	Bipolar Uncovered	My Mean Apps	Information	$0.99	1.02
20	Bipolar Explained	KoolAppz	Information	$4.69	1.0
21	Living with Bipolar Disorder	KoolAppz	Information	$1.29	1.0
23	Bipolar Disorder Symptoms	WE HELP YOU	Information	$0.00	
24	Bipolar Disorder Help	Appnok.co	Information	$1.26	1.0
26	BIPOLAR DISORDER UNCOVERED	IDJ Group	Information	$0.00	
28	What Is Bipolar Disorder	WE HELP YOU	Information	$0.00	
31	Psych Central	Liviant LLC	Information	$0.00	2.0
32	Free Mood Tracker	Invicta Trading and Promotion HB	Symptom monitoring	$0.00	1.3
39	Your.MD's Symptom Checker	Your.MD	Information	$0.00	

44	Health Tips	JD Star	Information	$0.00	2.2
49	Personal Psychology Tests	Darren Gates	Screening and assessment	$0.99	1.0
50	BipolarTracker Pro	ToTheHand, LLC	Symptom monitoring	$1.92	
56	iMoodJournal	Inexika Inc	Symptom monitoring	$0.99	1.3
58	Personal Progress Tracker	SoundMindz.Org	Symptom monitoring	$0.00	2.5
60	Moodlytics, Smart Mood Tracker	AnantApps by Avinashi	Symptom monitoring	$0.00	
62	Moodtrack Diary: Mood Tracker	Matthew Windwer	Symptom monitoring	$0.00	
63	1-800-therapist	1-800-therapist	Information	$0.00	1.0
64	Primary School Assessments	Darren Gates	Screening and assessment	$0.99	1.0
65	Smiley Calendar	3 ACORN Technologies, LLC	Symptom monitoring	$0.00	1.7
69	Bipol-App	Healthcare Learning Smile-o	Symptom monitoring	$0.00	
70	In Flow - Mood Diary	AITA LTD	Symptom monitoring	$0.00	1.19
72	Preschooler Assessments	Darren Gates	Screening and assessment	$0.99	1.0
75	Health Guide	Juan Beltran & Juan Herna	Information	$0.00	2.0
76	RxWiki	RxWiki, Inc	Information	$0.00	
78	MHF	Together For Change	Community support	$0.00	
80	How Are You - Mood Tracker	Quantum Lab Co.	Symptom monitoring	$13.82	1.61
81	Yoga for you	Yashendu Goswami	Information	$0.00	1.1
82	dailyRx	dailyRx, Inc	Information	$0.00	

83	WhatsMyM3	M3 Information	Screening and assessment	$0.00	1.9
87	Personal Depression Journal	Monitor My Health, LLC	Symptom monitoring	$2.22	1.1
90	Depression 101 by WAGmob	WAGmob (Simple 'n Easy)	Information	$2.14	2.5
95	Mood O Scope : Mood Tracker	XLabz Technologies Pvt Ltd	Symptom monitoring	$0.00	2.02
97	Mental Health Maintenance	Dnvgoods	Information	$0.00	1.0
105	Family	mhapps development	Information	$0.00	
106	MoodiModo BETA - Mood Tracker	The Abolitionist Project	Symptom monitoring	$0.00	1.53
109	Mental Health Disorders	Cool Help Guide	Information	$0.00	
114	Mood Tracker By: CTHF	Cheryl T. Herman Foundation	Symptom monitoring	$0.00	1.1
122	Mental Health At Work	eMETA Learning Solution	Information	$0.00	2.0
129	Your Child's Social Health	Darren Gates	Screening and assessment	$0.99	1.0
138	Control	mhapps development	Information	$0.00	
143	Activity record table / free	YabuSoft	Symptom monitoring	$0.00	1.1
164	Medical disease FAQ	Honey Computing Service	Information	$10.67	1.1
166	Activity record table	YabuSoft	Symptom monitoring	$1.70	1.0
167	Daily Mood	Kaizen Dev	Symptom monitoring	$0.00	1.3
168	Mental Health Maintenance	Bizzap	Information	$1.67	
177	Mood diary - depression	Julia Bechmann	Symptom monitoring	$2.96	1.1
179	Mood diary - depression Lite	Julia Bechmann	Symptom monitoring	$0.00	1.1
328	iMoodJournal -mood journal	Inexika Inc.	Symptom monitoring	$2.49	1.54

329	Bipolar Disorder Connect	Alliance Health Networks, Inc	Community support	$0.00	3.4
330	Optimism	Optimism Apps Pty Ltd	Symptom monitoring	$0.00	2.58
332	Bipolar Disorder Uncovered	AppWarrior	Information	$1.29	1.0
333	Moody Me - Mood Diary and Tracker	Medhelp	Symptom monitoring	$0.00	1.31
336	Bipolar Bear	Mark Braden	Awareness	$1.29	1.0
339	How Are You - depression, bipolar and mood	Quantum Lab Co.	Symptom monitoring	$16.99	2.83
345	In Flow - Mood and Emotion Diary	AITA LIMITED	Symptom monitoring	$0.00	2.5
348	Mood O Scope	XLabz Technologies Pvt. Ltd.	Symptom monitoring	$0.00	1.1
351	MoodLog	Markus Schopfer	Symptom monitoring	$0.00	1.0
353	Bipol-App	Healthcare Learning Company Ltd	Symptom monitoring	$0.00	
356	iMind & Mood	Samuel Lundin	Screening and assessment	$2.49	1.1
357	Better Mood Tracker - A Quantified Self Research Tool	Alexander Stone	Symptom monitoring	$0.00	1.3
358	iCouch CBT	iCouch Inc.	Treatment	$4.99	2.0
375	MHF	Neil Morris	Community support	$0.00	1.3
376	Personal Mood Chart Journal	Monitor My Health, LLC	Symptom monitoring	$3.79	1.1
378	MentalChecker	DuxSolutions	Screening and assessment	$1.29	1.0
393	WhatsMyM3	M-3 Information, LLC	Screening and assessment	$1.29	1.7
403	How was your mood	DevJockeys	Symptom	$1.29	1.1

			monitoring		
404	iBipolar	Michael Mahon	Information	$2.49	1.0
407	BipolarTrack	Gioel Asuni	Symptom monitoring	$2.49	3.0
409	SleepNotes - Lifestyle administrator in your pockets.	Masahiro Taniuchi	Symptom monitoring	$2.49	1.08
417	MoodWatcher	Samuel Fremaux	Symptom monitoring	$1.29	1.1
